# Impact of Shenfu injection on a composite of organ dysfunction development in critically ill patients with coronavirus disease 2019 (COVID-19): A structured summary of a study protocol for a randomized controlled trial

**DOI:** 10.1186/s13063-020-04677-5

**Published:** 2020-08-24

**Authors:** Zong-Yu Wang, Shou-Zhi Fu, Liang Xu, Shu-Sheng Li, Ke-Jian Qian, Xian-Di He, Guo-Chao Zhu, Liang-Hai Li, Jun Zhang, Wen-Fang Li, Bing-Yu Qin, Chen-Liang Zhou, Peng-Lin Ma

**Affiliations:** 1 Department of Intensive Care, Peking University Third Hospital, 49 North Garden Road, Haidian District, Beijing, 100191 P. R. China; 2 Department of Intensive Care Unit/Emergency, Wuhan Third Hospital, Wuhan university, Wuhan, Hubei P. R. China; 3 Department of Critical Care Medicine, Wuchang Hospital, Wuhan, Hubei P. R. China; 4 Department of Emergency and Critical Care Medicine, Tongji Hospital, Tongji Medical College, Huazhong University of Science and Technology, Wuhan, Hubei P. R. China; 5 Department of Critical Care Medicine, The First Affiliated Hospital of Nanchang University, Nanchang, Jiangxi P. R. China; 6 Department of Intensive Care Unit, The First Affiliated Hospital of Bengbu Medical College, Bengbu, Anhui P. R. China; 7 Department of Critical Care Medicine, The Affiliated Hospital of Jianghan University, Wuhan, Hubei P. R. China; 8 Department of Intensive Care Unit, Jingzhou Central Hospital, The Second Clinical Medical College, Yangtze University, Jingzhou, Hubei P. R. China; 9 Department of Critical Care Medicine, Wuhan Hospital of Traditional Chinese Medicine, Wuhan, Hubei P. R. China; 10 Department of Emergency and Critical Care, Changzheng Hospital, Naval Medical University, Shanghai, P. R. China; 11 Department of Critical Care Medicine, Henan Provincial People’s Hospital, Zhengzhou, Henan P. R. China; 12 Intensive Care Medicine, Eastern Campus, Renmin Hospital of Wuhan University, Wuhan, Hubei P. R. China

**Keywords:** COVID-19, Shenfu injection, randomised controlled trial, protocol

## Abstract

**Objectives:**

This study aims to determine the protection provided by Shenfu injection (a traditional Chinese medicine) against development of organ dysfunction in critically ill patients with coronavirus disease 2019 (COVID-19).

**Trial design:**

This study is a multicenter, randomized, controlled, open-label, two-arm ratio 1:1, parallel group clinical trial.

**Participants:**

The patients, who are aged from 18 to 75 years old, with a confirmed or suspected diagnosis of severe or critical COVID-19, will be consecutively recruited in the study, according to the guideline on diagnosis and treatment of COVID-19 (the 7^th^ version) issued by National Health Commission of the People’s Republic of China.

Exclusion criteria include pregnant and breastfeeding women, atopy or allergies to Shenfu Injection (SFI), severe underlying disease (malignant tumor with multiple metastases, uncontrolled hemopathy, cachexia, severe malnutrition, HIV), active bleeding, obstructive pneumonia caused by lung tumor, severe pulmonary interstitial fibrosis, alveolar proteinosis and allergic alveolitis, continuous use of immunosuppressive drugs in last 6 months, organ transplantation, expected death within 48 hours, the patients considered unsuitable for this study by researchers.

The study is conducted in 11 ICUs of designated hospitals for COVID-19, located in 5 cities of China.

**Intervention and comparator:**

The enrolled patients will randomly receive 100 ml SFI (study group) or identical volume of saline (control group) twice a day for seven consecutive days. Patients in the both groups will be given usual care and the necessary supportive therapies as recommended by the latest edition of the management guidelines for COVID-19 (the 7^th^ version so far).

**Main outcomes:**

The primary endpoint is a composite of newly developed or exacerbated organ dysfunction. This is defined as an increase in the sequential organ failure assessment (SOFA) score of two or more, indicating sepsis and involvement of at least one organ. The SOFA score will be measured for the 14 days after enrolment from the baseline (the score at randomization).

The secondary endpoints are shown below:

• SOFA score in total

• Pneumonia severity index score

• Dosage of vasoactive drugs

• Ventilation free days within 28 days

• Length of stay in intensive care unit

• Total hospital costs to treat the patient

• 28-day mortality

• The incidence of adverse drug events related to SFI

**Randomisation:**

The block randomization codes were generated by SAS V.9.1 for allocation of participants in this study. The ratio of random distribution is 1:1. The sealed envelope method is used for allocation concealment.

**Blinding (masking):**

The patients and statistical personnel analyzing study data are both blinded. The blinding of group assignment is not adopted for the medical staff.

**Numbers to be randomised (sample size):**

This study is expected to recruit 300 patients with COVID-19, (150 in each group).

**Trial Status:**

Protocol version 2.0, February 15, 2020.

Patient recruitment started on February 25, and will end on August 31, 2020.

**Trial registration:**

Chinese Clinical Trial Registry: ChiCTR2000030043. Registered February 21, 2020, http://www.chictr.org.cn/showprojen.aspx?proj=49866

**Full protocol:**

The full protocol is attached as an additional file, accessible from the *Trials* website (Additional file 1). In the interest in expediting dissemination of this material, the familiar formatting has been eliminated; this letter serves as a summary of the key elements of the full protocol.

## Supplementary information

**Additional file 1.** Full Study Protocol.

## Data Availability

P.L.M and Z.Y.W will have access to the final trial dataset. The dataset will be available after being authorized by the corresponding author on reasonable request. The contact e-mail address is mapenglin1@163.com.

