# Supplementary material for: Impact of Shenfu injection on a composite of organ dysfunction development in critically ill patients with coronavirus disease 2019 (COVID-19): A structured summary of a study protocol for a randomized controlled trial
Source: Trials. 2020 Aug 24;21:738. doi: 10.1186/s13063-020-04677-5 (PMC7443851; doi:10.1186/s13063-020-04677-5)
Supplement: Supplementary file 1 — Additional file 1. Full Study Protocol. [file 13063_2020_4677_MOESM1_ESM.doc]

**参附注射液治疗新型冠状病毒重症肺炎：一项多中心、随机对照、开放标签临床研究**

| **试验负责单位**： | 北京大学第三医院 |
| --- | --- |
| **试验参加单位：** | 全国12家医院（见附件4） |
| **试验方案设计：** | 多中心、随机对照、开放标签临床研究 |
| **日期：** | 2020年2月15日 |

研究方案摘要

| 负责单位 | 北京大学第三医院 |
| --- | --- |
| 研究设计 | 多中心、随机、对照、开放标签临床研究 |
| 研究目的 | 通过以下观察，评价参附注射液治疗新型冠状病毒所致重症肺炎的疗效和安全性。  1、评价用药后14天内新发器官功能损害及对肺炎严重指数（PSI）的影响。  2、观察用药后14天内器官衰竭序贯评分（SOFA）、28天内无机械通气时间、ICU住院天数、住院总费用以及28天病死率。  3、研究期间出现的药物不良事件。 |
| 药物名称 | 参附注射液（国药准字Z20043117） |
| 用法用量 | 参附注射液100ml，用等量生理盐水稀释后静脉滴注，1天2次。对严格限制液体量的患者（ARDS或心衰），可以通过深静脉泵入输注参附注射液。 |
| 疗程 | 7天 |
| 注意事项 | 参附注射液不能代替常规治疗措施，必须在常规治疗基础上同时使用。 |
| 受试对象 | 符合入选标准，不符合排除标准的新型冠状病毒感染的重症肺炎患者。 |
| 疗效指标 | PSI、14天内出现新发器官功能损害的百分率下降，器官衰竭序贯评分降低，机械通气时间缩短，ICU住院天数减少，28天病死率改善以及住院总费用降低。 |
| 安全指标 | 实验室动态监测患者血液常规、出凝血、肝脏及肾脏功能。 |
| 样本数量 | 300例 |

**临床研究流程图**

| **研究阶段** | **随机化前** | **治疗期1** | **治疗期2** | **治疗期3** | **观察期** | **随访1** |
| --- | --- | --- | --- | --- | --- | --- |
| **评估次序** | **第1次** | **第2次** | **第3次** | **第4次** | **第5次** | **第6次** |
| **观察时间点** | **用药前** | **用药1天** | **用药3天** | **用药7天** | **用药14天** | **用药28天** |
| **病例入选** | **√** |  |  |  |  |  |
| **基础疾病** | **√** |  |  |  |  |  |
|  | **医学评分评价** | | | | |  |
| **PSI评分（或其他评分系统评分）** | **√** | **√** | **√** | **√** | **√** |  |
| **SOFA评分** | **√** | **√** | **√** | **√** | **√** |  |
|  | **实验室检查及预后安全评估** | | | | |  |
| **生命体征** | **√** | **√** | **√** | **√** | **√** |  |
| **胸部影像学评价** | **√** |  |  |  |  |  |
| **血气分析** | **√** | **√** | **√** | **√** | **√** |  |
| **血管活性药物用量** | **√** | **√** | **√** | **√** | **√** |  |
| **血常规** | **√** | **√** | **√** | **√** | **√** |  |
| **肝、肾、出凝血功能** | **√** | **√** | **√** | **√** | **√** |  |
| **安全性评价** | **√** | **√** | **√** | **√** | **√** |  |
| **生存情况** | **√** | **√** | **√** | **√** | **√** | **√** |
| **机械通气时间** |  | **√** | | | | |
| **ICU住院时间** |  | **√** | | | | |
| **住院总花费** |  | **√** | | | | |
| **合并用药** | **√** | | | | |  |
| **给药** |  | **√** | | |  |  |

**参附注射液治疗新型冠状病毒重症肺炎：一项多中心、随机对照、开放标签临床研究**

**一、立题依据**

2019新型冠状病毒属于β属冠状病毒，主要经呼吸道飞沫传播，亦可通过接触传播，人群普遍易感，老年人及有基础疾病者感染后病情较重。该疾病临床表现以发热、乏力、干咳为主，少数患者伴有鼻塞、流涕、腹泻等症状。重型病例多在一周后出现呼吸困难，严重者快速进展为急性呼吸窘迫综合征、脓毒症休克、难以纠正的代谢性酸中毒和出凝血功能障碍。值得注意的是重型、危重型患者病程中可为中低热，甚至无明显发热。

目前尚无预防新型冠状病毒的专属性疫苗和有效杀灭新型冠状病毒的特效药物。针对该病毒感染所致肺炎的治疗，以对症治疗为主，包括一般支持治疗和氧疗等。对于重型和危重型病例，则在对症治疗的基础上，积极防治并发症，治疗基础疾病，预防继发感染，及时进行器官功能支持，包括呼吸支持、循环支持等。

中医药具有多靶点、集束化作用，对严重感染性疾病具有独特疗效。一些基础理论完善、临床定位明确、物质基础清楚、作用机制清晰的中药制剂在重症肺炎的治疗中已经显示出疗效优势，并积累了一定的循证医学证据。参附注射液是由红参、附片经现代制药工艺提取而成的中药制剂，药效物质是人参皂甙和乌头类生物碱。现代医学研究表明，参附注射液具有改善体循环、改善微循环、保护脏器细胞、增强细胞免疫等药理作用。（1）改善体循环方面，参附注射液通过调节心室重构后的TGF-β/Smads信号转导通路，降低Smad2和Smad3蛋白表达的同时增加Smad7的表达，改善心室结构和功能，减少心肌纤维化，降低血清脑尿钠肽(BNP)水平，保护心肌细胞。参附注射液能增加心肌收缩力，显著增加血管灌流量，提高平均动脉压，提高心排量、左室射血分数,改善大循环。（2）改善微循环方面，参附注射液能够增加全身毛细血管网开放数量、微动脉管径和血流速度；增加微血管流动指数，增加灌注血管密度和比例，改善小微血管和大微血管中的微循环血流量，同时可以提高氧输送、氧耗、氧摄取率，改善组织氧代谢，降低血乳酸。（3）保护脏器细胞方面，参附注射液能够减少细胞游离钙浓度，防止钙超载，清除氧自由基，抑制脂质过氧化物产生。通过PI3K/Akt通路激活心肌eNOS而发挥心肌保护作用；提高Na^+^-K^+^-ATP酶和Ca^2+^-ATP酶活性而改善心肌能量代谢；通过调节Bcl-2/Bax，抑制半胱胺酸-天门冬胺酸酶(Caspase-3)活性而抑制缺氧/复氧诱导的心肌凋亡；通过抑制TNF-α、IL-6、IL-8等炎性细胞因子的过度产生而减轻炎症反应对细胞的伤害,改善线粒体超微结构从而发挥抗损伤作用。（4）增强细胞免疫方面，参附注射液能增加外周血中的CD4^+^和CD8^+^ T细胞数量以及在单核细胞中上调HLA-DR表达,增强细胞免疫。

基于以上药理作用开展的临床试验研究表明，参附注射液联合常规治疗方案对重症肺炎患者血压、心排血指数、血管外肺水肿均有明显的改善作用，对患者的血液流变学有确切的效果，可减少血管活性药物使用量，改善内皮细胞功能，保护重要器官。因此，该药物被《中西医联合治疗社区获得性肺炎专家共识（2014 版）》等指南和共识推荐为重症肺炎治疗用药。

基于前期的基础医学研究和临床医学研究结果，《新型冠状病毒感染的肺炎诊疗方案》推荐参附注射液为重症期治疗用药。然而，新型冠状病毒作为新发现的致病原，其所致重症肺炎与其它已知致病原所致重症肺炎或有不同。有必要深入研究参附注射液对新型冠状病毒所致重症肺炎的治疗效果和安全性，以使该疾病的治疗方案更加完善。

**二、试验目的**

通过以下观察，评价参附注射液治疗新型冠状病毒所致重症肺炎的疗效和安全性。

1、对新发器官功能损害以及肺炎严重指数（PSI）的改善作用。

2、对预后的影响：用药后器官衰竭序贯评分（SOFA）变化、无机械通气时间、ICU住院天数、28天病死率以及住院总费用等。

3、研究期间出现的药物相关不良事件。

**三、试验设计**

1、试验类型：多中心、随机、对照、开放标签临床研究

2、对照方法：空白对照

3、试验样本量：按使发病14天内PSI绝对升高/绝对降低值（即入ICU时的PSI值和14天内PSI最高/最低值的差）变化20% 计算，总样本量为300例，试验组与对照组各150例。

4、多中心：在国内11家新型冠状病毒诊治定点医院的重症病房进行（附表4）。

**四、入选标准**

1、新型冠状病毒感染的肺炎确诊病例以及疑似病例（依据《新型冠状病毒感染的肺炎诊疗方案》诊断标准）（见附件3）

2、符合临床分型为“重型”和“危重型”（依据《新型冠状病毒感染的肺炎诊疗方案》）（见附件3）

3、18≤年龄≤75周岁

**五、排除标准**

1、孕妇、哺乳期妇女

2、过敏体质，或对参附注射液及其成分过敏者

3、影响生存的严重基础疾病，包括：未控制已经多处转移不能切除的恶性肿瘤、血液病、恶液质、活动性出血、严重营养不良、HIV等

4、肺部肿瘤致阻塞性肺炎、严重肺间质纤维化、肺泡蛋白沉积症、过敏性肺泡炎者

5、近 6个月内持续使用免疫抑制剂，或器官移植者

6、预计48小时内死亡者

7、负责研究者认为不适宜作为本试验研究对象的患者。

**六、终止和退出标准**

有下述情况时医师可中止研究试验对象，并且记录时间、原因：

1、研究对象或者直系家属要求退出本试验；

2、出现严重的不良事件；

3、其他特殊原因导致受试者中断试验。

**七、全面终止标准**

1、临床研究方案实施中发生重要偏差，难以评价药物疗效；

2、国家药品监督管理局或者相关行政部门提出终止本试验；

3、遇到不可抵抗力使研究无法再继续。

**八、治疗方案**

1、试验药品：参附注射液（国药准字Z20043117）。

2、用药方法与注意事项

⑴ 给药方法

| 组别 | 治疗药物 | 用法用量 | 给药时间 |
| --- | --- | --- | --- |
| 治疗组 | 常规治疗+参附 | 参附注射液100ml，用等量生理盐水稀释后静脉滴注，1天2次。对严格限制液体量的患者（ARDS或心衰），可以通过深静脉泵入输注参附注射液。 | 7天 |
| 对照组 | 常规治疗 |  |

注： 未死亡病例至少保证给药满7天,并在研究总结中记录用药情况。

深静脉泵入法输注100mL参附注射液，2小时泵完

⑵ 配药注意事项

* 使用100ml生理盐水加入100ml参附注射液。

* 与其它注射药物同时使用时，要进行生理盐水冲管。

* 本品禁止与其它注射药物配伍使用。

⑶ 注意事项

参附注射液不能代替常规治疗药物以及措施，必须在基础治疗同时使用。

3、常规治疗

参考最新版《新型冠状病毒感染的肺炎诊疗方案》给予对症治疗，积极防治并发症，治疗基础疾病，预防继发感染，及时进行器官功能支持，具体包括呼吸支持、循环支持、电解质平衡，以及抗菌、抗病毒及必要的血液制品等治疗（见附件3）。

**九、观察项目**

1、背景资料

1. 人口学资料：性别、年龄、身高、体重和民族
2. 基础疾病

2、有效性指标

- 1. 主要指标

发病14天内新发器官功能损害 (即SOFA任何一个器官单项得分变化增加≥2)发生率。

- 1. 次要指标：
     1. 器官衰竭序贯评分
     2. PSI评分

c. 血管活性药物用量

d. 28天内无机械通气时间

e. ICU住院时间

f. 住院总花费

g. 28天生存情况

**十、不良事件的观察**

**㈠、不良事件的记录**

对研究期间可能出现的心动过速、皮疹、呼吸困难等过敏反应，头晕、头痛、恶心呕吐、呃逆、震颤等不良事件，如实填写记录表，记录不良事件的发生时间、严重程度、持续时间、采取的有效措施和转归。

**㈡、安全性评定**

研究过程中发生的所有不良事件均依据CTCAE v5.0进行严重程度判定。

（1）1级：轻度；无症状或轻微；仅为临床或诊断所见；无需治疗；

（2）2级：中度；需要较小、局部或非侵入性治疗；与年龄相当的工具性日常生活活动受限；

（3）3级：严重或者具重要医学意义但不会立即危及生命；导致住院或者延长住院时间；致残；自理性日常生活活动受限；

（4）4级：危及生命；需要紧急治疗；

（5）5级：死亡。

**㈢、与药物因果关系判断**

1、不良反应判断的因果判断指标

①开始用药时间与可疑不良反应出现有无合理的先后关系；

②可疑不良反应是否符合该药品已知的不良反应类型；

③可疑不良反应能否用合并药作用、病人的临床状况或其他疗法的影响来解释；

④停药或减量后，可疑不良反应是否消失或减轻；

⑤再次接触可疑药品后，是否重新出现同样的反应。

因果判定标准：根据以上5条判断指标顺序判定。

不良反应因果判断

| 判断结果 | 判断指标 | | | | |
| --- | --- | --- | --- | --- | --- |
|  | ① | ② | ③ | ④ | ⑤ |
| 肯定有关 | + | + | - | + | + |
| 可能有关 | + | + | - | + | ？ |
| 无法确定 | + | + | ± | ± | ？ |
| 可能无关 | + | - | ± | ± | ？ |
| 无关 | - | - | + | - | - |

说明：+肯定、-否定、±难以肯定或否定、？情况不明。

2、根据上表，判定以下5级不良事件与药品的关系

1-肯定有关、2-可能有关、3-无法确定、4-可能无关、5-无关。

不良反应发生率的计算以1+2+3的病例总数作为分子，全部可供不良反应评价的入选病例作为分母。

**㈣、不良事件的处理**

1、报告方法：发生任何不良事件，如患者的主观不适及实验室检测异常，均需认真对待，仔细分析，立即采取措施保护受试者的安全。

2、处理程序：详细记录，并根据情况复测，记录其持续、转归、消失等情况。

3、严重不良事件的处理

根据所出现的症状做相应的处理，并在原始病历上详细记录。

4、随访未缓解的不良事件

所有不良事件都应当追踪，直到得到妥善解决或病情稳定。

**十一、数据管理**

考虑到研究疾病的特殊性，本项目将采用纸质病例报告表。

1、研究者根据受试者的原始观察记录，将数据及时、完整、正确、清晰地记载到病例报告表中。

2、监查员根据疫情情况在合适的时候对项目数据进行核查。确认所有病例报告表填写正确、完整，并与原始资料一致。如有错误和遗漏，及时要求研究者改正。修改时需保持原有记录清晰可见，改正处须经研究者签名并注明日期。

**十二、统计学方法**

**㈠ 数据集的选择**

1、全数据集（Full-text Analysis Set，FAS）：所有经随机化入组，至少使用一次研究药物、并至少进行过一次用药后疗效评估的病例，构成本研究的FAS人群。FAS人群中疗效相关部分的缺失数据将采用之前最后一次观测数据结转的方法进行补充。FAS将用于全部分析。

2、符合方案数据集（Per-Protocol Set, PPS）：PPS的标准及其人群将在数据盲态核查时最终确定，至少包括以下几个标准：(1)符合试验方案规定的入选标准；(2)完成全部计划访视；(3)试验期间未使用可能影响疗效评价的药物或治疗。PPS是本次研究疗效评价的次要人群。

3、安全性分析数据集（Safety Set，SS）：所有经随机化分组，至少使用一次研究药物、且具有用药后安全性评价数据的病例，构成本研究的安全性分析人群。

**㈡、统计分析计划**

1、统计分析将采用SAS9.4统计分析软件进行计算。最终确定的统计分析计划书应有主要研究者、统计学家、申办者的签字。

2、所有的统计检验均采用双侧检验，P值小于或等于0.05将被认为所检验的差别有统计意义。

3、进行组内治疗前后及组间的疗效指标比较分析。计量资料将采用均数±标准差进行统计描述。计量资料采用t检验比较组间差异，与筛选期基础值进行比较，采用配对Mixed比较组内前后差异。计数资料采用频数（构成比）进行统计描述。治疗前后组间的疗效差异采用秩和检验进行比较；疗效指标PSI评分、序贯器官衰竭(SOFA)评分采用方差分析固定效应模型进行检验。新发器官功能损害发生率采用卡方检验。

4、安全性分析：采用卡方检验比较两组不良事件发生率，并列表描述本次试验所发生的不良事件；比较实验室检查结果在试验前后正常/异常的变化情况以及发生异常改变时与试验药物的关系。

**十三、质量控制与质量保证**

1、试验方案确定：试验方案由所有参加本临床试验的研究者共同讨沦、协商，达成一致意见修改后，报送伦理委员会审批。

2、实验室的质控措施：各参加临床试验医院实验室建立统一实验检测指标、标准操作规程和质量控制程序。

3、研究者资格：参加临床试验的研究者必须具有临床试验的专业特长、资格和能力，经过资格审查，人员要求相对固定。

4、临床试验开始前培训：通过临床试验前培训使研究人员对于临床试验方案及其各指标具休内涵充分理解和认识。

5、实验室检查的异常判断标准，以检查单位的正常参考范围为准。

6、临床试验中所有观察结果和发现都应加以核实，以保证数据的可靠性，确保临床试验中各项结论来源于原始数据，在临床试验和数据处理阶段均有相应的数据管理措施。

7、针对可能发生的脱落，积极采取措施，控制病例脱落率在20％以内。

8、研究者根据受试者的原始观察记录，保证将数据正确(数据与患者的实际情况相符）、完整（不应有漏项）、清晰（字迹工整、易于辨认）、及时地录入病例报告表。

9、所有患者病历报告表由北京大学第三医院专人统一收集，并在固定位置保存，不经研究负责人同意任何人不得接触研究资料。

**十四、参考文献**

1、新型冠状病毒感染的肺炎诊疗方案（试行第五版）

2、2019 ATS/IDSA临床实践指南：成人社区获得性肺炎的诊断和治疗

3、中国成人社区获得性肺炎诊断和治疗指南（2016年版）

4、参附注射液急重症临床应用专家共识

5、中西医联合治疗社区获得性肺炎专家共识(2014版)

**附件**1：

| **肺炎严重指数（PSI）评分表** | | |
| --- | --- | --- |
| **姓名： 性别： 年龄： 岁 病室： 床号： 床**  **住院号：** | | |
| **统计** | **评估分数** | **实际分数** |
| 男 | 年龄值 |  |
| 女 | 年龄值-10 |
| 常住养老院 | +10 |  |
| **基础疾病** | | |
| 肿瘤疾病 | +30 |  |
| 肝脏疾病 | +20 |  |
| 充血性心力衰竭 | +10 |  |
| 脑血管疾病 | +10 |  |
| 肾脏疾病 | +10 |  |
| **生命体征异常体征** | | |
| 意识状态改变 | +20 |  |
| 呼吸≥30次/分 | +20 |  |
| 收缩压＜90mmHg | +20 |  |
| 体温＜35℃或≥40℃ | +15 |  |
| 脉搏≥125次/分 | +10 |  |
| **实验室和影像学表现** | | |
| 动脉PH＜7.35 | +30 |  |
| 血尿素氮≥9mmol/L | +20 |  |
| 血钠＜130mmol/L | +20 |  |
| 血糖≥14mmol/L | +10 |  |
| 红细胞压积（Hct）＜30% | +10 |  |
| PaO_2_＜60mmHg（或指氧饱和度＜90%） | +10 |  |
| 胸腔积液 | +10 |  |
| **总分合计** | |  |

**附件2**：

| **序贯器官衰竭(SOFA)评分表** | | | | |
| --- | --- | --- | --- | --- |
| **姓名： 性别： 年龄： 岁 病室： 床号： 床**  **住院号：** | | | | |
|  | **测量/评分值** | **评分标准** | **赋分** | **得分** |
| **呼吸系统**  （氧合指数） |  | ≥400 | 0 |  |
|  |  | ＜400 | 1 |  |
|  |  | ＜300 | 2 |  |
|  |  | ＜200+机械通气（无创/有创） | 3 |  |
|  |  | ＜100+机械通气（无创/有创） | 4 |  |
| **凝血系统**  （血小板计数） |  | ≥150 | 0 |  |
|  |  | ＜150 | 1 |  |
|  |  | ＜100 | 2 |  |
|  |  | ＜50 | 3 |  |
|  |  | ＜20 | 4 |  |
| **肝脏**  （胆红素umol/L） |  | ＜20 | 0 |  |
|  |  | 20~32 | 1 |  |
|  |  | 33~101 | 2 |  |
|  |  | 102~204 | 3 |  |
|  |  | ＞204 | 4 |  |
| **心血管系统**  药物剂量  （ug/kg•min） |  | Map≥70mmHg | 0 |  |
|  |  | Map＜70 mmHg | 1 |  |
|  |  | 多巴胺≤5  或多巴酚丁胺任何剂量 | 2 |  |
|  |  | 多巴胺＞5  或去甲肾上腺素≤0.1 | 3 |  |
|  |  | 多巴胺＞15  或去甲肾上腺素＞0.1 | 4 |  |
| **中枢神经系统**  （GCS评分) |  | 15 | 0 |  |
|  |  | 13~14 | 1 |  |
|  |  | 10~12 | 2 |  |
|  |  | 6~9 | 3 |  |
|  |  | ＜6 | 4 |  |
| **肾脏功能**  （肌酐umol/L） |  | ＜110 | 0 |  |
|  |  | 110~170 | 1 |  |
|  |  | 171~299 | 2 |  |
|  |  | 300~440或者尿量＜500ml/d | 3 |  |
|  |  | ＞440或者尿量＜200ml/d | 4 |  |
| **总分合计** | | | |  |
| 备注:1.每日评估时应采取每日最差值 | | | | |

**附件3：**

**《新型冠状病毒感染的肺炎诊疗方案（试行第五版）》临床诊断**

| 湖北以外省份：  （一）疑似病例。  结合下述流行病学史和临床表现综合分析：  1.流行病学史  （1）发病前14天内有武汉市及周边地区，或其他有病例报告社区的旅行史或居住史；  （2）发病前14天内与新型冠状病毒感染者（核酸检测 阳性者）有接触史；  （3）发病前14天内曾接触过来自武汉市及周边地区，或来自有病例报告社区的发热或有呼吸道症状的患者；  （4）聚集性发病。  2.临床表现  （1）发热和／或呼吸道症状；  （2）具有上述肺炎影像学特征；  （3）发病早期白细胞总数正常或降低，或淋巴细胞计数减少。  有流行病学史中的任何一条，且符合临床表现中任意2无明确流行病学史的2条，符合临床表现中的3条。  （二）确诊病例。  疑似病例，具备以下病原学证据之一者：  1.呼吸道标本或血液标本实时荧光RT-PCR检测新型冠状病毒核酸阳性；  2.呼吸道标本或血液标本病毒基因测序，与已知的新型冠状病毒高度同源。  湖北省：  （一）疑似病例。  结合下述流行病学史和临床表现综合分析：  1.流行病学史  （1）发病前14天内有武汉市及周边地区， 或其他有病例报告社区的旅行史或居住史；  （2）发病前14天内与新型冠状病毒感染者（核酸检测阳性者）有接触史；  （3）发病前14天内曾接触过来自武汉市及周边地区，或来自有病例报告社区的发热或有呼吸道症状的患者；  （4）聚集性发病。  2.临床表现  （1）发热和／或呼吸道症状；  （2）发病早期白细胞总数正常或降低，或淋巴细胞计数减少。  有流行病学史中的任何一条或无流行病学史，且同时符合临床表现中2条。  （二）临床诊断病例。  疑似病例具有肺炎影像血特征者。  （三）确诊病例。  临床诊断病例或疑似病例，具备以下病原学证据之一者：  1.呼吸道标本或血液标本实时荧光RT-PCR检测新型冠状病毒核酸阳性；  2.呼吸道标本或血液标本病毒基因测序，与已知的新型冠状病毒高度同源。 |
| --- |

**《新型冠状病毒感染的肺炎诊疗方案（试行第五版）》临床分型**

| （一）轻型  临床症状轻微，影像学未见肺炎表现。  （二）普通型  具有发热、呼吸道等症状，影像学可见肺炎表现。  （三）重型  符合下列任何一条：  1、呼吸窘迫，RR≥30 次/分；  2、静息状态下，指氧饱和度≤93%；  3、动脉血氧分压（PaO2）/吸氧浓度（FiO2）≤300mmHg （1mmHg=0.133kPa）。  （四）危重型  符合以下情况之一者：  1、出现呼吸衰竭，且需要机械通气；  2、出现休克；  3、合并其他器官功能衰竭需ICU 监护治疗。 |
| --- |

**《新型冠状病毒感染的肺炎诊疗方案（试行第五版）》治疗方案**

| （一）根据病情确定治疗场所。  1.疑似及确诊病例应当在具备有效隔离条件和防护条件的定点医院隔离治疗，疑似病例应单人单间隔离治疗，确诊病例可多人收治在同一病室。  2.危重型病例应当尽早收入ICU治疗。  （二）一般治疗。  1.卧床休息，加强支持治疗，保证充分热量；注意水、电解质平衡，维持内环境稳定；密切监测生命体征、指氧饱和度等。  2.根据病情监测血常规、尿常规、CRP、生化指标（肝酶、心肌酶、肾功能等）、凝血功能，动脉血气分析，胸部影像学等。有条件者可行细胞因子检测。  3.及时给予有效氧疗措施，包括鼻导管、面罩给氧和经鼻高流量氧疗。  4.抗病毒治疗：目前没有确认有效的抗病毒治疗方法。可试用α-干扰素雾化吸入（成人每次500万U或相当剂量，加入灭菌注射用水2ml，每日2次）、洛匹那韦/利托那韦（200 mg/50 mg，每粒）每次2粒，每日2次，或可加用利巴韦林（成人首剂4g，次日每8小时一次，每次1.2g，或8mg/kg iv.每8小时一次）。要注意洛匹那韦/利托那韦相关腹泻、恶心、呕吐、肝功能损害等不良反应，同时要注意和其他药物的相互作用。  5.抗菌药物治疗：避免盲目或不恰当使用抗菌药物，尤其是联合使用广谱抗菌药物。  （三）重型、危重型病例的治疗。  1.治疗原则：在对症治疗的基础上，积极防治并发症，治疗基础疾病，预防继发感染，及时进行器官功能支持。  2.呼吸支持：  （1）氧疗：重型患者应当接受鼻导管或面罩吸氧，并及时评估呼吸窘迫和（或）低氧血症是否缓解。  （2）高流量鼻导管氧疗或无创机械通气：当患者接受标准氧疗后呼吸窘迫和（或）低氧血症无法缓解时，可考虑使用高流量鼻导管氧疗或无创通气。若短时间（1-2小时）内病情无改善甚至恶化，应及时进行气管插管和有创机械通气。  （3）有创机械通气：采用肺保护性通气策略，即小潮气量（4-8ml/kg理想体重）和低吸气压力（平台压<30cmH2O）进行机械通气，以减少呼吸机相关肺损伤。较多患者存在人机不同步，应当及时使用镇静以及肌松剂。  （4）挽救治疗：对于严重ARDS患者，建议进行肺复张。在人力资源充足的情况下，每天应进行12小时以上的俯卧位通气。俯卧位通气效果不佳者，如条件允许，应尽快考虑体外膜肺氧合（ECMO）。  3.循环支持：充分液体复苏的基础上，改善微循环，使用血管活性药物，必要时进行血流动力学监测。  4.其他治疗措施  可根据患者呼吸困难程度、胸部影像学进展情况，酌情短期内（3～5日）使用糖皮质激素，建议剂量不超过相当于甲泼尼龙1～2mg/kg/日，应当注意较大剂量糖皮质激素由于免疫抑制作用，会延缓对冠状病毒的清除；可静脉给予血必净100ml/次，每日2次治疗；可使用肠道微生态调节剂，维持肠道微生态平衡，预防继发细菌感染；可采用恢复期血浆治疗；对有高炎症反应的危重患者，有条件可以考虑使用体外血液净化技术。  患者常存在焦虑恐惧情绪，应当加强心理疏导。  （四）中医治疗。  本病属于中医疫病范畴，病因为感受疫戾之气，各地可根据病情、当地气候特点以及不同体质等情况，参照下列方案进行辨证论治。  1.医学观察期  临床表现1：乏力伴胃肠不适  推荐中成药：藿香正气胶囊（丸、水、口服液）  临床表现2：乏力伴发热  推荐中成药：金花清感颗粒、连花清瘟胶囊（颗粒）、疏风解毒胶囊（颗粒）、防风通圣丸（颗粒）  2.临床治疗期  （1）初期：寒湿郁肺  临床表现：恶寒发热或无热，干咳，咽干，倦怠乏力，胸闷，脘痞，或呕恶，便溏。舌质淡或淡红，苔白腻，脉濡。  推荐处方：苍术15g、陈皮10g、厚朴10g、藿香10g、草果6g、生麻黄6g、羌活10g、生姜10g、槟郎10g  （2）中期：疫毒闭肺  临床表现：身热不退或往来寒热，咳嗽痰少，或有黄痰，腹胀便秘。胸闷气促，咳嗽喘憋，动则气喘，舌质红，苔黄腻或黄燥，脉滑数。  推荐处方：杏仁10g、生石膏30g、瓜蒌30g、生大黄6g（后下）、生炙麻黄各6g、葶苈子10g、桃仁10g、草果6g、槟郎10g、苍术10g  推荐中成药：喜炎平注射剂，血必净注射剂  （3）重症期：内闭外脱  临床表现：呼吸困难、动辄气喘或需要辅助通气，伴神昏，烦躁，汗出肢冷，舌质紫暗，苔厚腻或燥，脉浮大无根。  推荐处方：人参15g、黑顺片10g（先煎）、山茱萸15g，送服苏合香丸或安宫牛黄丸  推荐中成药：血必净注射液、参附注射液、生脉注射液  （4）恢复期：肺脾气虚  临床表现：气短、倦怠乏力、纳差呕恶、痞满，大便无力，便溏不爽，舌淡胖，苔白腻。  推荐处方：法半夏9g、陈皮10g、党参15g、炙黄芪30g、茯苓15g、藿香10g、砂仁6g（后下） |
| --- |

**附件4：**

**参与试验的定点收治医院**

| **编号** | **省份** | **医院名称** |
| --- | --- | --- |
| 1 | 安徽 | 蚌埠医学院第一附属医院 |
| 2 | 河南 | 河南省人民医院 |
| 3 | 江西 | 南昌大学第一附属医院 |
| 4 | 湖北 | 荆州市中心医院 |
| 5 | 湖北 | 武汉市第三医院 |
| 6 | 湖北 | 武昌医院 |
| 7 | 湖北 | 武汉市第六医院 |
| 8 | 湖北 | 华中科技大学同济医学院附属同济医院 |
| 9 | 湖北 | 武汉市人民医院 |
| 10 | 湖北 | 武汉市中医医院 |
| 11 | 上海 | 长征医院 |


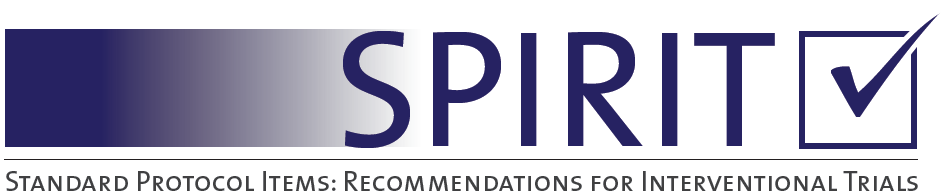


SPIRIT 2013 Checklist: Recommended items to address in a clinical trial protocol and related documents*

| Section/item | ItemNo | Description |
| --- | --- | --- |
| **Administrative information** | | |
| ✓Title | 1 | Descriptive title identifying the study design, population, interventions, and, if applicable, trial acronym |
| ✓Trial registration | 2a | Trial identifier and registry name. If not yet registered, name of intended registry |
|  | 2b | All items from the World Health Organization Trial Registration Data Set |
| ✓Protocol version | 3 | Date and version identifier |
| ✓Funding | 4 | Sources and types of financial, material, and other support |
| ✓Roles and ✓responsibilities | 5a | Names, affiliations, and roles of protocol contributors |
|  | 5b | Name and contact information for the trial sponsor |
|  | 5c | Role of study sponsor and funders, if any, in study design; collection, management, analysis, and interpretation of data; writing of the report; and the decision to submit the report for publication, including whether they will have ultimate authority over any of these activities |
|  | 5d | Composition, roles, and responsibilities of the coordinating centre, steering committee, endpoint adjudication committee, data management team, and other individuals or groups overseeing the trial, if applicable (see Item 21a for data monitoring committee) |
| Introduction |  |  |
| ✓Background and rationale | 6a | Description of research question and justification for undertaking the trial, including summary of relevant studies (published and unpublished) examining benefits and harms for each intervention |
|  | 6b | Explanation for choice of comparators |
| ✓Objectives | 7 | Specific objectives or hypotheses |
| ✓Trial design | 8 | Description of trial design including type of trial (eg, parallel group, crossover, factorial, single group), allocation ratio, and framework (eg, superiority, equivalence, noninferiority, exploratory) |
| Methods: Participants, interventions, and outcomes | | |
| ✓Study setting | 9 | Description of study settings (eg, community clinic, academic hospital) and list of countries where data will be collected. Reference to where list of study sites can be obtained |
| ✓Eligibility criteria | 10 | Inclusion and exclusion criteria for participants. If applicable, eligibility criteria for study centres and individuals who will perform the interventions (eg, surgeons, psychotherapists) |
| ✓Interventions | 11a | Interventions for each group with sufficient detail to allow replication, including how and when they will be administered |
|  | 11b | Criteria for discontinuing or modifying allocated interventions for a given trial participant (eg, drug dose change in response to harms, participant request, or improving/worsening disease) |
|  | 11c | Strategies to improve adherence to intervention protocols, and any procedures for monitoring adherence (eg, drug tablet return, laboratory tests) |
|  | 11d | Relevant concomitant care and interventions that are permitted or prohibited during the trial |
| ✓Outcomes | 12 | Primary, secondary, and other outcomes, including the specific measurement variable (eg, systolic blood pressure), analysis metric (eg, change from baseline, final value, time to event), method of aggregation (eg, median, proportion), and time point for each outcome. Explanation of the clinical relevance of chosen efficacy and harm outcomes is strongly recommended |
| ✓Participant timeline | 13 | Time schedule of enrolment, interventions (including any run-ins and washouts), assessments, and visits for participants. A schematic diagram is highly recommended (see Figure) |
| ✓Sample size | 14 | Estimated number of participants needed to achieve study objectives and how it was determined, including clinical and statistical assumptions supporting any sample size calculations |
| ✓Recruitment | 15 | Strategies for achieving adequate participant enrolment to reach target sample size |
| **Methods: Assignment of interventions (for controlled trials)** | | |
| ✓Allocation: |  |  |
| Sequence generation | 16a | Method of generating the allocation sequence (eg, computer-generated random numbers), and list of any factors for stratification. To reduce predictability of a random sequence, details of any planned restriction (eg, blocking) should be provided in a separate document that is unavailable to those who enrol participants or assign interventions |
| Allocation concealment mechanism | 16b | Mechanism of implementing the allocation sequence (eg, central telephone; sequentially numbered, opaque, sealed envelopes), describing any steps to conceal the sequence until interventions are assigned |
| Implementation | 16c | Who will generate the allocation sequence, who will enrol participants, and who will assign participants to interventions |
| ✓Blinding (masking) | 17a | Who will be blinded after assignment to interventions (eg, trial participants, care providers, outcome assessors, data analysts), and how |
|  | 17b | If blinded, circumstances under which unblinding is permissible, and procedure for revealing a participant’s allocated intervention during the trial |
| **Methods: Data collection, management, and analysis** | | |
| ✓Data collection methods | 18a | Plans for assessment and collection of outcome, baseline, and other trial data, including any related processes to promote data quality (eg, duplicate measurements, training of assessors) and a description of study instruments (eg, questionnaires, laboratory tests) along with their reliability and validity, if known. Reference to where data collection forms can be found, if not in the protocol |
|  | 18b | Plans to promote participant retention and complete follow-up, including list of any outcome data to be collected for participants who discontinue or deviate from intervention protocols |
| ✓Data management | 19 | Plans for data entry, coding, security, and storage, including any related processes to promote data quality (eg, double data entry; range checks for data values). Reference to where details of data management procedures can be found, if not in the protocol |
| ✓Statistical methods | 20a | Statistical methods for analysing primary and secondary outcomes. Reference to where other details of the statistical analysis plan can be found, if not in the protocol |
|  | 20b | Methods for any additional analyses (eg, subgroup and adjusted analyses) |
|  | 20c | Definition of analysis population relating to protocol non-adherence (eg, as randomised analysis), and any statistical methods to handle missing data (eg, multiple imputation) |
| **Methods: Monitoring** | | |
| ✓Data monitoring | 21a | Composition of data monitoring committee (DMC); summary of its role and reporting structure; statement of whether it is independent from the sponsor and competing interests; and reference to where further details about its charter can be found, if not in the protocol. Alternatively, an explanation of why a DMC is not needed |
|  | 21b | Description of any interim analyses and stopping guidelines, including who will have access to these interim results and make the final decision to terminate the trial |
| ✓Harms | 22 | Plans for collecting, assessing, reporting, and managing solicited and spontaneously reported adverse events and other unintended effects of trial interventions or trial conduct |
| ✓Auditing | 23 | Frequency and procedures for auditing trial conduct, if any, and whether the process will be independent from investigators and the sponsor |
| Ethics and dissemination | | |
| ✓Research ethics approval | 24 | Plans for seeking research ethics committee/institutional review board (REC/IRB) approval |
| ✓Protocol amendments | 25 | Plans for communicating important protocol modifications (eg, changes to eligibility criteria, outcomes, analyses) to relevant parties (eg, investigators, REC/IRBs, trial participants, trial registries, journals, regulators) |
| ✓Consent or assent | 26a | Who will obtain informed consent or assent from potential trial participants or authorised surrogates, and how (see Item 32) |
|  | 26b | Additional consent provisions for collection and use of participant data and biological specimens in ancillary studies, if applicable |
| ✓Confidentiality | 27 | How personal information about potential and enrolled participants will be collected, shared, and maintained in order to protect confidentiality before, during, and after the trial |
| ✓Declaration of interests | 28 | Financial and other competing interests for principal investigators for the overall trial and each study site |
| ✓Access to data | 29 | Statement of who will have access to the final trial dataset, and disclosure of contractual agreements that limit such access for investigators |
| ✓Ancillary and post-trial care | 30 | Provisions, if any, for ancillary and post-trial care, and for compensation to those who suffer harm from trial participation |
| ✓Dissemination policy | 31a | Plans for investigators and sponsor to communicate trial results to participants, healthcare professionals, the public, and other relevant groups (eg, via publication, reporting in results databases, or other data sharing arrangements), including any publication restrictions |
|  | 31b | Authorship eligibility guidelines and any intended use of professional writers |
|  | 31c | Plans, if any, for granting public access to the full protocol, participant-level dataset, and statistical code |
| Appendices |  |  |
| ✓Informed consent materials | 32 | Model consent form and other related documentation given to participants and authorised surrogates |
| [Not applicable] Biological specimens | 33 | Plans for collection, laboratory evaluation, and storage of biological specimens for genetic or molecular analysis in the current trial and for future use in ancillary studies, if applicable |

*It is strongly recommended that this checklist be read in conjunction with the SPIRIT 2013 Explanation & Elaboration for important clarification on the items. Amendments to the protocol should be tracked and dated. The SPIRIT checklist is copyrighted by the SPIRIT Group under the Creative Commons “[Attribution-NonCommercial-NoDerivs 3.0 Unported](http://www.creativecommons.org/licenses/by-nc-nd/3.0/)” license.
